# Estimation of cancer incidence and mortality attributable to alcohol drinking in china

**DOI:** 10.1186/1471-2458-10-730

**Published:** 2010-11-25

**Authors:** Hao Liang, Jianbing Wang, Huijuan Xiao, Ding Wang, Wenqiang Wei, Youlin Qiao, Paolo Boffetta

**Affiliations:** 1 Department of Cancer Epidemiology, Cancer Institute, Chinese Academy of Medical Sciences, Beijing, China; 2 Departments of Epidemiology and Statistics, Institute of Basic Medical Sciences Chinese Academy of Medical Sciences & School of Basic Medicine, Peking Union Medical College, Beijing, China; 3 Department of Nutrition and Food Hygiene, West China School of Public Health, Sichuan University, Chengdu, China; 4 The Tisch Cancer Institute, Mount Sinai School of Medicine, New York, USA; 5 International Prevention Research Institute, Lyon, France

## Abstract

**Background:**

Cancer constitutes a serious burden of disease worldwide and has become the second leading cause of death in China. Alcohol consumption is causally associated with the increased risk of certain cancers. Due to the current lack of data and the imperative need to guide policymakers on issues of cancer prevention and control, we aim to estimate the role of alcohol on the cancer burden in China in 2005.

**Methods:**

We calculated the proportion of cancers attributable to alcohol use to estimate the burden of alcohol-related cancer. The population attributable fraction was calculated based on the assumption of no alcohol drinking. Data on alcohol drinking prevalence were from two large-scale national surveys of representative samples of the Chinese population. Data on relative risk were obtained from meta-analyses and large-scale studies.

**Results:**

We found that a total of 78,881 cancer deaths were attributable to alcohol drinking in China in 2005, representing 4.40% of all cancers (6.69% in men, 0.42% in women). The corresponding figure for cancer incidence was 93,596 cases (3.63% of all cancer cases). Liver cancer was the main alcohol-related cancer, contributing more than 60% of alcohol-related cancers.

**Conclusions:**

Particular attention needs to be paid to the harm of alcohol as well as its potential benefits when making public health recommendations on alcohol drinking.

## Background

Cancer constitutes a serious burden of disease worldwide as well as in China. Ranked as the second leading cause of death in China, cancer accounts for 22.32% of deaths, behind only cerebrovascular disease (22.45% of total deaths)[1]. Alcohol consumption is causally associated with the increased risk of certain cancers [2]. In China, alcohol drinking has a long history and continues to be an important part of life and culture. Alcohol consumption by Chinese citizens has increased in recent decades following rapid economic development that entailed cultural and behavioral changes [3,4]. As a strategic program of "Healthy China 2020" is being developed [5], an estimate of the burden of alcohol-related cancer is imperative for guiding policymakers on issues of cancer prevention and control. The aim of our study is to estimate the cancer burden which is attributable to alcohol drinking in the year 2005.

## Methods

Our study is an evidence-based and consistent evaluation and is part of a project called "attributable causes of cancer in China", aimed at estimating the role of demonstrated carcinogenic factors, including smoking, chronic infection, nutritional and anthropometric factors, occupational exposures, and hormonal factors on the cancer burden in China. Specific cancer sites which are causally associated with alcohol drinking include oral cavity, larynx, pharynx, esophagus, liver, female breast as well as colorectum [2].

Estimation of the attributable causes of cancers was performed by calculating the proportion of specific cancers occurring in China in 2005 attributable to specific risk factors. The proportion of cancers in the total population that can be attributed to a risk factor is called the population attributable fraction (PAF), and corresponds to the proportional reduction in the population disease or mortality that would occur if exposure to the risk factor was modified according to an alternative exposure scenario.

### Definition of exposure

Alcohol consumption was defined as drinking alcohol on at least 12 occasions during the past 12 months. We did not take into account the type of alcohol (beer, wine, distilled spirit) or drinking patterns (regular vs binge drinking) as this information was not included in our data.

### Data used for estimating relative risk (RR) of alcohol drinking and cancers

Data on the RR of alcohol and cancer was collected through a search from 1990 to 2008, in the databases Pubmed, Ovid, China National Knowledge Infrastructure (CNKI), VIP Information and authoritative publications or data from the Ministry of Health in China [1,6-8]. The search words included "meta-analysis", "case-control study", "cohort study", "alcohol drinking", "alcohol consumption", "China" and the names of specific cancers. Inclusion criteria of studies were: 1) Data obtained in the past 10 years; 2) Contained relative risk (RR) or odds ratio (OR) and corresponding 95% confidence intervals (CI); 3) Meta-analyses or large-scale surveys of representative samples of China were given the highest priority, followed by non-representative samples of China, or meta-analyses from Asian or Western countries. 4) Definition of RRs of alcohol-related cancers was consistent with our study.

For each study, we extracted details on study design, population, number of subjects (cases, controls or cohort size), sex of the population considered, and confounders allowed for in the analysis, RR estimates for categories of alcohol consumption and the corresponding 95% CI.

The RRs of alcohol drinking and cancers are listed in Table 1. We excluded female breast cancer, for which no excess risk from alcohol has been shown in Chinese studies [8,9], and derived the RRs of alcohol drinking and cancer of esophagus [10], colorectum [11], and liver [12] from meta-analysis studies in China. RRs of oral cavity and pharyngeal cancer were obtained from a prospective cohort study of 61,320 Singaporean Chinese people aged 45-74 years [13]. However, since RR estimates for other cancers were not available from studies of Chinese or other Asian countries, the RR of laryngeal cancer was derived from a recent systematic literature review of cohort studies [2]. The same RRs were applied to both men and women (Table 1). The counterfactual exposure scenario consisted of no exposure to alcohol at all.

**Table 1:** Relative Risk for Alcohol drinking in China

Cancer Site	RR	95% CI	Design	Population	Reference
Oral cavity Pharynx	2.3	1.2,4.3	Cohort Study	Singaporean Chinese	Friborg 2007[13]
Esophagus	1.51	1.18,1.3	Meta-Analyses	China	Yu 2003[10]
Colorectum	1.06	1.25,1.81	Meta-Analyses	China	Chen 2003[11]
§Liver	1.87	1.36,2.57	Meta-Analyses	China	Pei 2008[12]
Larynx	1.24	0.91,1.24	Meta-Analyses	Worldwide	WCRF/AICR 2007[2]

§Liver cancer refers to hepatocellular cancer

### Data used for exposure prevalence

We assumed a latency of 15 years between alcohol exposure and its effect on cancer risk, due to past patterns of exposure. Thus, data on alcohol drinking prevalence were therefore obtained in the1990 s to calculate the PAF of alcohol on cancers in 2005.

Data on the prevalence of alcohol consumption were taken from the "1991 National Hypertension Survey of China", which covered mainland China's 30 provinces, autonomous regions, and municipalities with the use of a multistage design of random cluster sampling. 949,539 people over 15 years of age were recruited by survey, and the overall prevalence of alcohol drinking was 17.94% (35.09% for men and 2.58% for women) [14].

We conducted a sensitivity analysis in which we used data on prevalence of alcohol drinking in 2002 derived from the large-scale National Nutrition Survey, which included over 240,000 subjects older than 15 years from 31 provinces, autonomous regions, and municipalities, excluding Hong Kong, Macao and Taiwan [7]. The prevalence of alcohol drinking in 2002 was higher than in 1991, with 39.6% among men and 4.5% among women.

### Cancer mortality and incidence data

Cancer mortality data were collected from by the Third National Death Cause Retrospective Survey China (Table 2) [1]. In brief, this survey was conducted in 160 randomized counties and 53 high-risk areas for cancer from 2004 to 2005, and results were extrapolated to the rest of the country. Cancer incidence data were estimated by using the Mortality and Incidence (M/I) ratio and cancer deaths from the Third National Death Cause Retrospective Survey. The M/I ratio was derived from the data of 32 regional cancer registry sites between 2003 and 2004 in China. The M/I ratio was calculated using Poisson regression to adjust for age and gender [6].

**Table 2:** Number of deaths and cases of cancer in 2005 in China

Site	ICD 10	Male		Female	
			
		Deaths	Cases	Deaths	Cases
Oral cavity	C00-08	4838	10187	2324	5344
Pharynx	C09-10 C12-14	2516	3670	872	1570
Esophagus	C15	131685	163361	58548	73228
Colon	C18	20596	42596	16570	34830
Rectum	C19-20	36138	67409	26028	46475
§Liver	C22	222127	247106	83250	86107
Larynx	C32	8935	15270	2622	3158
All cancers *	C00-96 except C44	1136877	1527747	654736	1050414

* All cancers but non-melanoma skin

§Liver cancer refers to hepatocellular cancer

### Calculation of PAF

The PAF can be calculated as a function of the relative risk (RR) of cancer associated with alcohol drinking and the prevalence of alcohol drinking (P) of a given population. The following formula was used [15]:

$$\text{PAF}=\frac{\text{P}\times \left(\text{RR-1}\right)}{\left[\text{P}\times \left(\text{RR-1}\right)\right]+\text{1}}$$

## Results

Alcohol drinking was responsible for 4.40% of all cancer deaths in China, with a total of 76,109 male deaths (6.69%) and 2,774 female deaths (0.42%) (Table 3 and 4). 3.63% of all cancer cases were attributable to alcohol drinking, with a total of 90,386 male cases (5.92%) and 3210 female cases (0.31%). Liver cancer was the main alcohol-related cancer, contributing over 60% of all alcohol-related cancer deaths.

**Table 3:** Number of cancer deaths and cases attributable to alcohol drinking by sex in China in 2005, based on 1991 drinking habits

Cancer site	Male			Female		
		
	PAF (%)	Deaths	Cases	PAF (%)	Deaths	Cases
Oral cavity, Pharynx	31.33	2304	4341	3.25	104	224
Esophagus	15.18	19989	24797	1.30	760	951
Colorectum	2.06	1170	2268	0.15	66	126
§Liver	23.39	51592	57794	2.20	1828	1890
Larynx	7.77	694	1186	0.62	16	19
Total		76109	90386		2774	3210
% of all cancers		6.69%	5.92%		0.42%	0.31%

§Liver cancer refers to hepatocellular cancer

**Table 4:** Number of cancer deaths and cases attributable to alcohol drinking in China in 2005, based on 1991 drinking habits

Cancer site	Number of deaths	% of all cancers (%)	Number of cases	% of all cancers (%)
Oral cavity, Pharynx	2407	0.13	4565	0.18
Esophagus	20749	1.16	25748	1.00
Colorectum	1236	0.07	2394	0.09
§Liver	53779	3.00	59684	2.32
Larynx	710	0.04	1205	0.05
Total	78881	4.40	93596	3.63

§Liver cancer refers to hepatocellular cancer

The sensitivity analysis based on 2002 alcohol-drinking prevalence data resulted in a fraction of cancer deaths attributable to alcohol equal to 7.36% for men and 0.73% for women; the fraction of cancer incident cases attributable to alcohol was 6.50% for men and 0.53% for women (Table 5).

**Table 5:** Number of cancer deaths and cases attributable to alcohol drinking by sex in China based on 2002 drinking habits

Cancer site	Male			Female		
		
	PAF (%)	Deaths	Cases	PAF (%)	Deaths	Cases
Oral cavity, Pharynx	33.98	2499	4709	5.53	177	382
Esophagus	16.80	22126	27449	2.24	1314	1643
Colorectum	2.32	1317	2553	0.27	115	219
§Liver	25.62	56918	63318	3.77	3136	3244
Larynx	8.68	775	1325	1.07	28	34
Total		83635	99354		4770	5522
% of all cancers		7.36%	6.5%		0.73%	0.53%

§Liver cancer refers to hepatocellular cancer

## Discussion

This is the first study specifically aimed at evaluating the burden of alcohol-related cancer in China. We found that a total of 78,881 cancer deaths (4.40%) and 93,596 cancer cases (3.63%) were attributable to alcohol use in China. Liver cancer was the dominant alcohol-related cancer in China, contributing more than 60% of all alcohol-related cancer deaths among women and men. PAFs for women were lower than those for men, because of the much lower prevalence of alcohol consumption among women in China. A sensitivity analysis based on the two largest national surveys in 1991 and 2002 showed an increased prevalence of drinking during more recent years in China, particularly among women. Although it is more likely that the cancers occurring in 2005 resulted from alcohol consumption in the early 1990 s, the results of the sensitivity analysis suggest that the burden of alcohol-related cancer in China is likely to increase in forthcoming years.

Boffetta and colleagues [16] estimated that 3.5% of all cancers deaths (5.1% in men, 1.3% in women) and a similar proportion of cancer cases were attributable to alcohol drinking worldwide in 2002, also, Danaei and colleagues [17] estimated that 5% of all cancer deaths in low and middle-income countries were attributable to alcohol use in 2001. Our estimates are comparable to these earlier data. Interestingly, stomach cancer represents a special case. Although no consistent evidence has been found linking stomach cancer and alcohol consumption[18], this conclusion is based primarily on studies conducted in Europe and the Americas, while most relevant Chinese studies provide evidence of an association. In particular, a recent meta-analysis including 27 articles on alcohol drinking and stomach cancer published over a period of 15 years resulted in an overall RR of 2.03 [95% CI: 1.74-2.37] [19]. If we consider the association to be causal and apply the RR from this meta-analysis, we obtain a PAF for stomach cancer of 26.55% in men, corresponding to 56,510 deaths, and 2.59% in women (2,741 deaths). Adding these figures to the totals reported in Table 3 and 4 would increase the total proportion of cancer deaths attributable to alcohol drinking to 7.71% (6.78% for incidence). Given the potential importance of alcohol-related stomach cancer, it is important to clarify whether the association observed in Chinese studies is indeed causal or is instead due to confounding or other biases.

As one of the most common cancers in China, breast cancer has increased dramatically in both urban and rural areas over the past 30 years [20]. Despite many studies in Western countries that have established an association between alcohol consumption and the risk of breast cancer, a meta-analysis of the risk factors of breast cancer in Chinese women [9] and a large-scale study in 6 cities in China [8] reported no association of breast cancer with alcohol drinking. A similar conclusion was derived from studies of Japanese women: in a review of 11 epidemiological studies on alcohol drinking and breast cancer from Japan, a positive association was reported in only one of three cohort studies and two of eight case-control studies [21]. We therefore did not include breast cancer in our study. But if, as done for stomach cancer, we try to estimate the effect of alcohol on breast cancer, assuming it applies also to Chinese women, the result is 1% [22] based on a recent estimate of PAF done by WCRF, which corresponds to 1,416 female deaths in 2005.

Most studies have found no association between alcohol drinking and pancreatic cancer risk [2,23,24], although a few studies have recorded an increased risk. A recent meta-analysis [25] of 32 studies resulted in a relative risk (RR) of 0.92 (95% CI, 0.86-0.97) for moderate drinkers and 1.22 (95% CI, 1.12-1.34) for heavy drinkers, but the stratified analysis for Asia showed no association. Also, a meta-analysis of risk factors for pancreatic cancer in China with 1,889 cases and 10,304 controls reported no association using the same definition of alcohol drinking as our study [26]. Since tobacco smoking is a strong risk factor for pancreatic cancer, residual confounding cannot be ruled out in these studies. The available evidence for an association between pancreatic cancer and alcohol consumption is not convincing, so we did not take pancreatic cancer into account in our calculations.

According to our study, liver cancer is a dominant alcohol-related cancer in China with the highest mortality rate and PAF value, followed by oral cavity and pharynx cancers. For our study, we regard liver cancer as just hepatocellular cancer, without considering cholangiocarcinoma, which accounts for approximately 10% of liver cancers[27] but is not associated with alcohol [28,29]. Risk of liver cancer has strong synergistic effects with tobacco, hepatitis B virus and hepatitis C virus [30], especially in China where high prevalence of HPV and HCV infections is thought to be a dominant factor. The fact that RRs from meta-analyses in China were not adjusted for these factors might have resulted in an overestimate of the alcohol PAF for this disease.

With respect to head and neck cancers (oral cavity, pharynx, and larynx), although there is strong evidence of a causal association with alcohol drinking, the low incidence of these cancers in China resulted in a relatively small number of cancers attributable to alcohol. Other major risk factors for these cancers including tobacco smoking, HPV infection and dietary factors, such as low fruit and vegetable intake, have been well established [31].

Our results have several limitations. First, the RR of larynx cancer was obtained from a systematic literature review of cohort studies conducted in non-Chinese populations. Second, some of the original studies included in the meta-analyses may not have adjusted the risk estimates for alcohol for potential confounding factors, such as smoking for head and neck cancer, or HBV and HCV infection for liver cancer, leading to an overestimate of the effect of alcohol in our calculations. Third, the same RR was used for both genders, while differences in the strength of association may actually be present between genders, due to differences in average alcohol consumption by gender. Fourth, some biases need to be considered, including that data on cancer incidence were not available and were estimated based on mortality-to-incidence ratios, which may be subject to bias. However, most attributable cases were from lethal cancers, and the extrapolation from mortality data was not a great issue. Another possible bias is that the meta-analyses from which our data collected were made using data from case-control studies, which are generally considered more susceptible to recall and selection biases than cohort studies. In addition, misclassification of past alcohol drinking may lead to observation biases. Finally, we did not take into consideration the pattern of drinking (e.g. binge or regular drinking), or the type of beverage (e.g. beer, wine, distilled spirit) because of uncertainties in the quantification of the effect of such factors on cancer risk.

## Conclusions

Despite more than 60% of Chinese men and 90% of women reporting no alcohol drinking, alcohol consumption accounted for 4.40% of cancer deaths and 3.63% of cancer cases in China in 2005. Recently, a National Nutrition Survey with data from 2002 reported that alcohol drinking has increased by 17.3% compared with 1991 National Hypertension Survey [7], with an increase of 12.8% among men and 73.1% among women. It is likely that alcohol use and related cancers will continue to increase as a public health problem in China over the coming years. Although from the point of view of cancer prevention, the best level of alcohol consumption is zero, evidence shows that low and moderate (1-2 drinks/day) alcohol consumption might reduce the risk of cardiovascular disease. Since the important factor is the amount of ethanol consumed, control of heavy drinking remains the main target for cancer control. Also, particular attention needs to be paid regarding the potential harms of alcohol as well as its potential benefits when making public health recommendations on alcohol drinking. For example, the Dietary Guidelines for Chinese Residents (2007) recommends that those who do not drink should continue to avoid drinking alcohol, but people who already drink are recommended to limit consumption to no more than 30 grams of ethanol a day for men and 15 grams of ethanol per day for women [32]. As China is a country with a high incidence of cerebrovascular disease, total avoidance of alcohol, especially for current alcohol consumers, although optimal for cancer control, should not be recommended as a public health policy.

## Competing interests

The authors declare that they have no competing interests.

## Authors' contributions

HL participated in the design of the study and carried out the literature review, statistical analysis, and drafted the manuscript. JBW participated in the design of the study and helped in statistical analysis. HJX and DW helped in the literature review. WQW and YLQ participated in the design of the study and supervision. PB participated in the design of the study, helped in statistical analysis and drafting the manuscript as well as supervision. All authors read and approved the final manuscript.

## Pre-publication history

The pre-publication history for this paper can be accessed here:

http://www.biomedcentral.com/1471-2458/10/730/prepub
